# Association between maternal education and objectively measured physical activity and sedentary time in adolescents

**DOI:** 10.1136/jech-2015-205763

**Published:** 2016-01-22

**Authors:** Lauren B Sherar, Tom P Griffin, Ulf Ekelund, Ashley R Cooper, Dale W Esliger, Esther M F van Sluijs, Lars Bo Andersen, Greet Cardon, Rachel Davey, Karsten Froberg, Pedro C Hallal, Kathleen F Janz, Katarzyna Kordas, Susi Kriemler, Russell R Pate, Jardena J Puder, Luis B Sardinha, Anna F Timperio, Angie S Page

**Affiliations:** 1 National Centre for Sport and Exercise Medicine, School of Sport, Exercise and Health Sciences, Loughborough University, Loughborough, UK; 2 NIHR Leicester – Loughborough Diet, Lifestyle, and Physical Activity Biomedical Research Unit, Leicester, UK; 3 Centre for Exercise, Nutrition and Health Sciences/School of Social and Community Medicine, University of Bristol, Bristol, UK; 4 Norwegian School for Sport Science, Oslo, Norway; 5 MRC Epidemiology Unit, University of Cambridge, Cambridge, UK; 6 Centre for Exercise, Nutrition and Health Sciences/School for Policy Studies, University of Bristol, Bristol, UK; 7 Centre of Diet and Activity Research, Unicersity of Cambridge, Cambridge, UK; 8 Department of Sports Science and Clinical Biomechanics, University of Southern Denmark, Odense, Denmark; 9 Department of Movement and Sport Sciences, Ghent University, Ghent, Belgium; 10 Centre for Research & Action in Public Health, University of Canberra, Canberra, Australia; 11 Federal University of Pelotas, Pelotas, Brazil; 12 Department of Health and Human Physiology, Department of Epidemiology, University of Iowa, Iowa City, Iowa, USA; 13 School of Social and Community Medicine, University of Bristol, Bristol, UK; 14 Epidemiology, Biostatistics and Prevention Institute, University of Zürich, Zürich, Switzerland; 15 Department of Exercise Science at the University of South Carolina, South Carolina, USA; 16 Service of Endocrinology, Diabetes and Metabolism, University Hospital, CHUV, Lausanne, Switzerland; 17 Faculty of Human Kinetics, University of Lisbon, Lisbon, Portugal; 18 School of Exercise and Nutrition Sciences/Centre for Physical Activity and Nutrition Research, Deakin University, Melbourne, Victoria, Australia

**Keywords:** PHYSICAL ACTIVITY, SOCIAL INEQUALITIES, PAEDIATRIC

## Abstract

**Background:**

Investigating socioeconomic variation in physical activity (PA) and sedentary time is important as it may represent a pathway by which socioeconomic position (SEP) leads to ill health. Findings on the association between children's SEP and objectively assessed PA and/or sedentary time are mixed, and few studies have included international samples.

**Objective:**

Examine the associations between maternal education and adolescent's objectively assessed PA and sedentary time.

**Methods:**

This is an observational study of 12 770 adolescents (10–18 years) pooled from 10 studies from Europe, Australia, Brazil and the USA. Original PA data were collected between 1997 and 2009. The associations between maternal education and accelerometer variables were examined using robust multivariable regression, adjusted for a priori confounders (ie, body mass index, monitor wear time, season, age and sex) and regression coefficients combined across studies using random effects meta-analyses. Analyses were conducted in March 2014.

**Results:**

Adolescents of university educated mothers spent more time sedentary (9.5 min/day, p=0.005) and less time in light activity (10 min/day, p<0.001) compared with adolescents of high school educated mothers. Pooled analysis across two studies from Brazil and Portugal (analysed separately because of the different coding of maternal education) showed that children of higher educated mothers (tertiary vs primary/secondary) spent less time in moderate to vigorous PA (MVPA) (6.6 min/day, p=0.001) and in light PA (39.2 min/day: p<0.001), and more time sedentary (45.9 min/day, p<0.001).

**Conclusions:**

Across a number of international samples, adolescents of mothers with lower education may not be at a disadvantage in terms of overall objectively measured PA.

## Introduction

Socioeconomic position (SEP) is an important correlate of health and well-being. Studies show that in adults from developed countries, low SEP, measured through household income and education, is related to poor health and greater all-cause mortality.^1^ ^2^ Likewise, populations from developing countries who are socioeconomically disadvantaged in terms of income or education, tend to fare worse in terms of non-communicable diseases,^3^ although some epidemiological evidence suggests that the early adoption of unhealthy behaviours by newly socioeconomically advantaged populations may lead to an inverse association between education and health in some countries.^4^

Children growing up in low SEP families in developing countries are more likely to be overweight^5^ ^6^ and have greater risk for cardiovascular disease^2^ ^7^ and poor psychological health^8^ than children of a higher SEP, although the association may differ by country of origin.^9^ Socioeconomic variation in physical activity (PA) is important as it may represent an intermediary pathway by which SEP leads to poor health. In PA research, however, SEP is rarely used as a primary variable of interest, and is more frequently included to account for potential confounding effects. Within the relatively small body of literature that has directly examined associations between PA and SEP, findings are equivocal and tend to focus on middle/high income and/or developed countries.^10^ ^11^ A systematic review (which included papers from the USA, Asia, Canada, Oceania, Brazil, Europe (and 1 study that included 32 countries)) suggested that higher SEP was associated with higher levels of PA in adolescents.^11^ However, 42% of studies reported no association or a negative association. Reasons for these equivocal results are that previous studies^10–13^ have used (1) varying indicators of SEP, (2) subjective, self-reported measures of PA, which are known to have inherent biases,^14^ (3) varying domains (eg, active travel, leisure time) of PA. That said, a more consistent association has been shown between SEP and indicators of sedentary behaviour, such as self-reported television watching in Europe and the USA.^5^ ^10^ ^15–18^ Fewer studies have examined the association between objectively measured total sedentary time and SEP.

Objective measurement tools such as accelerometers, more accurately quantify PA and total sedentary time than self-reporting methods.^14^ However, even among studies using objective measures, findings on the association between SEP and PA^17^ ^19–22^ and/or sedentary time^15^ ^21^ ^23^ are mixed. Given the multiple proxy indicators (eg, maternal education, paternal education, household income, etc) and composite indicators (eg, National Statistics Socioeconomic classification) of SEP used in published literature, it is difficult to compare results across studies. Interpretation of the accelerometry literature is similarly challenging given the multitude of analytical methods/criteria used to generate outcome variables, such as criteria for establishing wear time versus non-wear, determining what constitutes a valid day/file and choice of intensity cut points. The recently compiled International Children's Accelerometry Database (ICAD)^24^ pooled and analysed raw accelerometer data using standardised methods to create comparable outcome variables across studies from Europe, Australia, Brazil and the USA. In addition, separate indicators of maternal education were pooled and recoded to create standardised maternal education categories across studies. Therefore, the ICAD affords the opportunity to investigate, for the first time, cross-sectional associations between maternal education, PA and time spent sedentary across a large number of international samples using standardised SEP and accelerometer data in a meta-analytical approach.

## Methods

### Study design

The ICAD (http://www.mrc-epid.cam.ac.uk) has been described in detail elsewhere along with dates of data collection.^24^ In brief, the eligibility criteria for inclusion in the ICAD were (1) PA data in the form of raw accelerometer (.dat) files from a version of a waist-worn Actigraph accelerometer (eg, 7164, 71256, GT1M1) on children aged 3–18 years, (2) accompanying data, at a minimum, of gender, age and measured height and weight. Formal data sharing agreements were established, and all partners consulted with their individual research boards to confirm sufficient ethical approval had been attained for contributing data.

### Participants

For the present study, the analytical sample (n=12 770) was restricted to children aged 10–18 years. This age range was chosen as it yields the largest number of participants with maternal education information. Data were taken from 10 studies, across eight countries: Australia (N=2: 2001/2004/2006; 2002–2003/2006), Brazil (2006–2007), Denmark (1997–1998 and 2003–2004), Estonia (1998–1999), Portugal (1999–2000), Switzerland (2005–2006), the UK (N=2: 2003–2007; 2006–2009) and the USA (2002–2006). Where longitudinal data was available, only baseline data was used.

### Maternal education

Owing to the variation in national education systems and categories used to record educational level (see online supplementary table S1), raw maternal education variables were standardised to enable comparisons across studies. To maintain meaningful interpretation, the studies were divided into two groups for analysis, and maternal education was standardised separately for each group. For eight of the studies, the raw education variables were recoded into the following three categories: (1) ‘high school’—no education, or completed some/all of high school, (2) ‘college’—completed some/all of college education (including vocational training), (3) ‘university’—completed some/all of a university degree (including graduate school). The reason for selecting these categories (rather than including a ‘not completed high school’ category, commonly seen in other literature) is because three studies did not collect data on not completing high school, and when they did few were placed into this category. Where data was collected on not completing high school (5 studies), different criteria/cut-offs were used. The raw education variable for the remaining two studies (Pelotas study of Brazilian children and The Portugal European Youth Heart Study (EYHS) of children from Madeira) recorded school attainment in years of education, and thus, no distinction could be made between vocational and university training. These studies were analysed separately on an educational scale defined as (1) primary school education and (2) secondary school/tertiary education. Online supplementary table S1 also indicates the number of years of education for each education category by study.

### Assessment of PA and sedentary time

Detailed description of the assessment of PA is available elsewhere.^24^ Briefly, all available accelerometer data from the ICAD project were reanalysed, to provide comparable PA and sedentary outcomes across studies, using specifically developed and commercially available software (KineSoft, V.3.3.20, Loughborough, UK; http://www.kinesoft.org). Data files were reintegrated to 60 s epochs and non-wear time was defined as 60 min of consecutive zeros allowing 2 min of non-zero interruptions.^25^ All children with ≥ 1 day with ≥500 min of wear time between 7:00 and midnight were included, in keeping with previous analyses.^26^ A more thorough discussion of the appropriateness of using a single day of wear is available elsewhere.^27^ Raw accelerometer-derived PA data were expressed as average counts per minute (CPM; ie, total counts per valid day, divided by monitor wear time per day). Time spent sedentary was defined as all minutes <100 CPM,^28^ and MVPA time as minutes >3000 CPM,^28^ ^29^ corresponding to about 4.6 metabolic equivalents.^28^

### Anthropometry

Height and weight was measured using standardised techniques within studies. Body mass index (BMI) was calculated as weight (kg)/height (m)^2^ and participants were categorised into normal weight, overweight and obese groups according to age-specific and sex-specific thresholds.^30^

### Statistical analysis

Descriptive results are expressed as mean and SD for continuous variables and percentages for categorical variables. Two-way analysis of variance (ANOVA) was used to explore possible interactions between sex and maternal education in relation to each PA outcome. Associations between CPM, light activity, moderate-to-vigorous PA (MVPA), and sedentary time were analysed using Pearson correlation. Robust linear regression was used to estimate the mean difference in PA outcomes between the maternal education categories within each study. Models were adjusted a priori for age, sex, season and monitor wear time, with the exception of CPM models, in which wear time is inherent in the derived variable. A binary classification was used as an indicator of season (based on daylight). Data collected in the Northern hemisphere were classified as either summer (data collected May–October) or winter (data collected November–April), and inverted for Southern hemisphere data.

Reprocessing the raw accelerometer data allowed measurement of the dependent and independent variables on the same scales, and thus, comparison of mean differences in PA between maternal education categories. Mean differences of the studies were combined to estimate an overall mean difference (ie, a combined estimate), and 95% CI between maternal education categories for each accelerometer outcome in turn by using random effects meta-analysis models weighted by the inverse of the variances (precision) of the mean differences. Heterogeneity among the study estimates was assessed by visual inspection of forest plots and by the I^2^ statistic.^31^ Heterogeneity between studies may be reduced through modelling relative rather than absolute differences in accelerometer outcomes between maternal educational categories. To explore this, analyses were repeated using within-study standardisation of the accelerometer outcomes. Last, in addition to controlling for the aforementioned confounders the analyses were repeated when (1) adjusting for BMI status (normal weight/overweight/obese) to assess whether the observed difference in PA and sedentary time between maternal education categories was independent of BMI status, (2) mutually adjusting for MVPA and sedentary time (ie, when MVPA was modelled as the main exposure, the analysis was adjusted for sedentary time and vice versa) to establish if differences in MVPA and sedentary time between maternal education categories were independent of each other. All analyses were conducted using Stata/SE V.11.2 in March 2014. Significance testing was two sided, and p values were used to assess the strength of evidence against the null hypotheses.

## Results

Ten ICAD studies representing 16 275 youths aged 10–18 years, met the inclusion criteria for the present analysis. Of this sample, 12 770 (78.5%) had at least 1 day of ≥500 min of valid accelerometer wear time and a self-reported measure of maternal education, and thus, formed the study population.

The mean age of included participants was 12.4 years (SD=1.4) and 66.9% were female. The average number of monitored days for these participants was 5.3, with a daily monitor wear time of 790 min (SD=78). Seventy-five per cent of the participants were normal weight, 18.2% overweight and 6.8% obese. Participant characteristics by maternal grouping and study are summarised in table 1.

**Table 1:** Characteristics of study populations (mean (SD)) by study and maternal education group

Study	Maternal education group	N	Age (year)	BMI (kg/m^2^)	% NW/OW/OB	Days of monitoring	Summer sample (%)	Monitor wear time (min/day)	CPM	Sedentary (min/day)	Light activity (min/day)	MVPA (min/day)
ALSPAC*	High School	2972	11.7 (0.2)	19.2(3.4)	77, 18, 5	6.1	52	769(59)	596 (196)	348 (71)	390 (67)	34.8 (20.6)
(UK)	College	691	11.7 (0.2)	19.1 (3.5)	79, 17, 4	6.1	51	773 (59)	591 (192)	353 (72)	391 (66)	34.5 (20.8)
	University	1195	11.7 (0.2)	18.6 (2.9)	84, 14, 2	6.2	52	779 (58)	577 (180)	370 (69)	380 (64)	34.7 (19.7)
CLAN	High School	461	11.5 (0.6)	19.9 (3.9)	71, 21, 8	5.5	26	781 (69)	673 (213)	335 (69)	413 (64)	38.7 (20.6)
(Australia)	College	83	11.5 (0.6)	20.4 (3.8)	66, 25, 9	5.6	28	791 (70)	651 (201)	339 (69)	419 (61)	36.5 (19.0)
	University	267	11.5 (0.6)	19.1 (3.3)	75, 22, 3	5.5	32	779 (69)	651 (202)	348 (72)	397 (61)	37.7 (19.7)
EYHS	High School	184	13.8 (2.5)	20 (3.4)	85, 14, 2	3.8	32	783 (75)	505 (312)	408 (133)	356 (112)	25.8 (24.8)
(Denmark)	College	182	13.5 (2.6)	19.6 (3.1)	85, 14, 1	4.1	27	792 (66)	485 (247)	413 (121)	363 (106)	25.3 (20.9)
	University	149	13 (2.7)	19 (2.9)	88, 12, 0	4.3	22	789 (61)	511 (264)	405 (128)	365 (112)	27.0 (22.8)
EYHS	High School	102	14.7 (1.9)	19.8 (2.7)	93, 7, 0	3.9	41	806 (65)	555 (241)	394 (104)	384 (91)	38.8 (25.7)
(Estonia)	College	105	14.8 (1.8)	20.1 (3)	88, 11, 1	3.8	29	811 (60)	578 (256)	375 (106)	409 (88)	38.0 (27.4)
	University	114	14.7 (1.8)	19.9 (2.8)	93, 5, 2	3.9	57	815 (53)	544 (228)	403 (96)	379 (83)	39.2 (26.1)
HEAPS	High School	426	11.3 (0.6)	19.8 (3.4)	65, 27, 8	4.9	31	760 (77)	659 (224)	325 (73)	404 (65)	37.1 (22.2)
(Australia)	College	77	11.3 (0.6)	20.7 (3.6)	56, 34, 10	5.0	29	778 (84)	629 (205)	342 (76)	406 (66)	35.4 (20.3)
	University	276	11.2 (0.6)	19.1 (3.2)	76, 17, 6	4.9	30	765 (76)	670 (193)	325 (68)	407 (66)	40.0 (20.8)
PEACH	High School	379	10.9 (0.4)	18.5 (3.2)	76, 20, 4	5.1	42	740 (74)	560 (170)	362.2 (70)	355 (60)	26.3 (16.3)
(UK)	University	173	10.9 (0.4)	17.6 (2.5)	88, 10, 2	5.8	66	757 (59)	552 (135)	382 (64)	351 (56)	27.9 (14.9)
Pelotas	Primary	370	13.3 (0.3)	20.3 (3.7)	77, 19, 4	3.8	52	932 (78)	420 (161)	516 (91)	391 (93)	23.5 (18.0)
(Brazil)	Secondary/Tertiary	86	13.7 (2.6)	20.7 (4.0)	73, 17, 10	3.8	48	917 (84)	339 (127)	557 (99)	342 (90)	17.3 (11.9)
EYHS	Primary	324	13.8 (2.6)	20.4 (3.6)	81, 14, 5	3.7	32	789 (76)	546 (235)	387 (104)	372 (94)	30.5 (23.5)
(Portugal)	Secondary/Tertiary	23	13.9 (2.7)	20.7 (3.6)	83, 13, 4	4.0	29	799 (66)	420 (178)	438 (90)	340 (92)	20.7 (20.0)
Project TAAG	High School	1250	13.4 (1.1)	22.8 (5.5)	62, 21, 17	4.8	2	788 (82)	391 (150)	447 (84)	328 (73)	16.0 (12.2)
(USA)	College	708	13.4 (1.0)	22.7 (5.5)	61, 23, 16	4.8	2	790 (79)	405 (146)	446 (77)	330 (73)	17.5 (12.5)
	University	1951	13.3 (1.0)	21.3 (4.6)	73, 18, 9	5.0	3	795 (79)	404 (143)	452 (83)	328 (71)	18.4 (13.0)
KISS	High School	56	11.3 (0.5)	18.4 (3)	78, 20, 2	6.3	100	908 (90)	683 (172)	399 (78)	465 (75)	50.0 (22.7)
(Switzerland)	College	104	11.2 (0.5)	18.2 (2.7)	85, 13, 2	6.7	89	919 (66)	628 (197)	436 (87)	444.5 (65)	45.2 (24.3)
	University	62	10.9 (0.5)	17.7 (2.7)	89, 10, 2	6.6	100	905 (82)	654 (164)	425 (83)	441 (57)	46.4 (20.0)

*See online supplementary material.

ALSPAC, Avon Longitudinal Study of Parents and Children; BMI, body mass index; CLAN, Children Living In Active Neighbourhoods; CPM, counts per minute; EYHS, European Youth Heart Study; HEAPS, Healthy Eating and Play Study; KISS, Kinder-Sportstudie Study; MVPA, moderate-to-vigorous physical activity; NW, normal weight; OB, obese; OW, overweight; PEACH, Personal And Environmental Associations With Children’s Health; Pelotas, Pelotas 1993 birth cohort; Project TAAG, Project Trial of Activity for Adolescent Girls.

**Figure 1:**
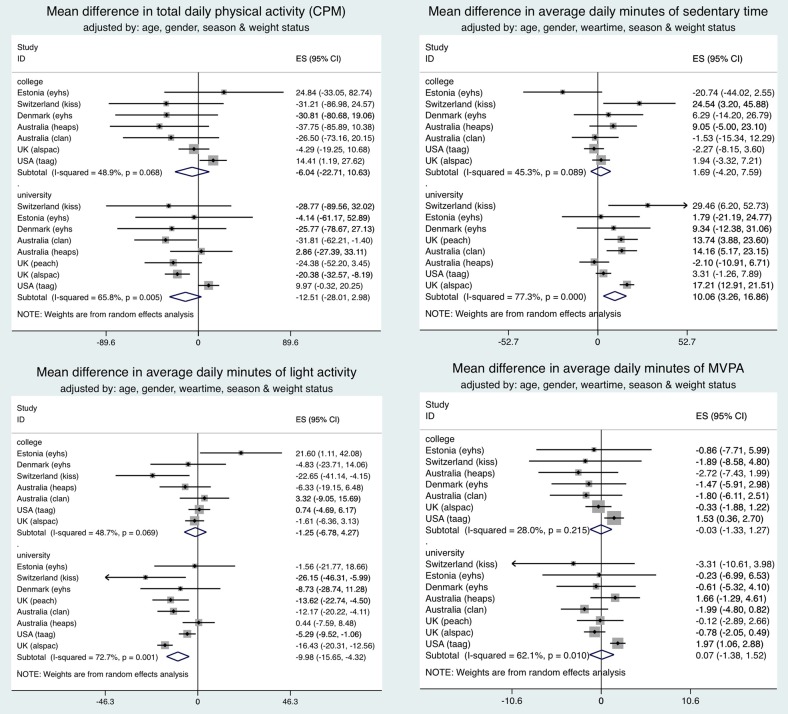
Forest plot showing mean differences, after adjustment for body mass index status, in physical activity variables between studies where maternal education defined as: high school (reference group), college and university.

Table 1 shows the average daily PA (CPM, minutes spent in light and MVPA), and time sedentary by study. After controlling for monitor wear, girls spent 55 more minutes sedentary compared to boys (mean: 406 min/day (SD=92.1) vs 351 min/day (SD=87.5), p<0.001). Girls accumulated 34 min less light activity (mean: 356 min/day (SD=74.7) vs 390 min/day (SD=71.5), p<0.001) and 20 min less in MVPA (mean: 22.6 min/day (SD=15.5) vs 42.1 min/day (SD=23.6), p<0.001) per day than boys. Two-way ANOVAs provided no evidence of interactions between sex and maternal education when considering their relationship with each PA outcome in turn. Consequently, data for boys and girls were combined, and models adjusted for sex. Inspection of forest plots and the I^2^ statistic with ‘high school’ as the reference group (figure 1) indicated that moderate to high levels of heterogeneity (I^2^>25%^32^) were present in estimates of the mean difference in PA outcomes by maternal education. Random-effects meta-analysis was used, due to these differences in the effect of maternal education across studies.

### Maternal education defined as high school, college and university: eight studies

For all PA outcomes (CPM, sedentary, light and MVPA), and after controlling for confounders (age, season, sex and monitor wear time (where appropriate)), no significant differences were found in the combined estimate (ie, overall mean difference across studies between maternal education categories) for children of college versus high school educated mothers (see online supplementary figure S1). However, significant differences in PA were found between children of university educated mothers compared with high school educated mothers. Adjustment for sedentary time in addition to the previously mentioned confounders resulted in a higher pooled estimate of MVPA (2.2 min, p<0.001) and lower heterogeneity (I^2^=0%) in children of university versus high school educated mothers.

Across all three maternal education groups, the only pooled estimates that were significant at the 0.05 level were time spent sedentary (9.5 min/day, p=0.005) and in light activity (−10.0 min/day, p<0.001) for children of university compared to high school educated mothers. For these pooled estimates there was a high level of heterogeneity within the study-specific estimates (sedentary: I^2^=76.8%; light: I^2^=71.9%), but high consistency in the point estimates either above or below the line representing ‘no effect’ (see online supplementary figure S1). Repeating the main analysis adjusting for BMI status as well as other confounders had no substantive impact on the results (see figure 1).

### Maternal education defined as primary, secondary and tertiary: two studies

**Figure 2:**
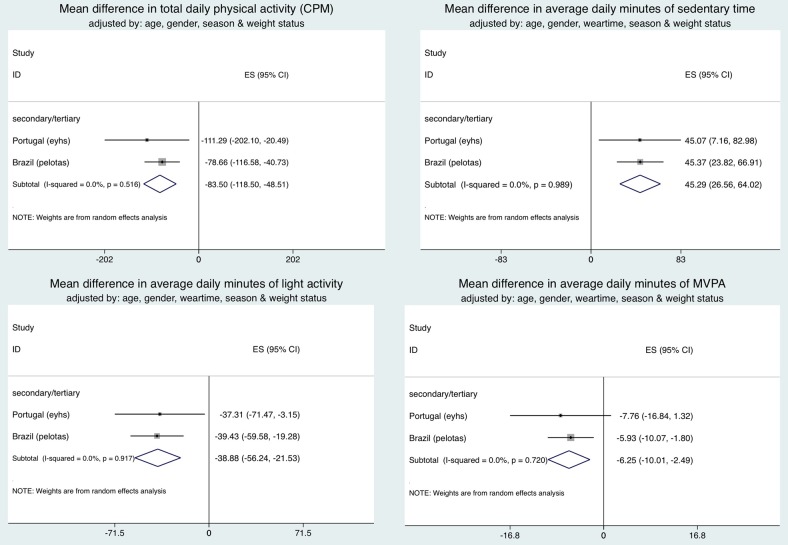
Forest plot showing mean differences, after adjustment for body mass index status, in physical activity variables between studies where maternal education defined as: primary (reference group), secondary and tertiary.

Findings within this group were similar to those observed within the main set of studies (see online supplementary figure S2). After controlling for confounders children of higher educated mothers (secondary/tertiary vs primary educated) on average participated in less MVPA (−6.6 min/day, p=0.001), light PA (−39.2 min/day, p<0.001) and CPM (−85.4 min/day, p<0.001) and spent more time sedentary (45.9 min/day, p<0.001). Repeating the main analyses adjusting for BMI status in addition to other confounders did not alter the results (figure 2).

## Discussion

Pooled analyses from the eight studies from the UK, Australia, Denmark, Estonia, the USA and Switzerland showed that adolescents of university educated mothers spent approximately 10 more minutes sedentary and approximately 10 less minutes in light activity per day compared with adolescents of high school educated mothers. In the two studies from Brazil and Portugal that were categorised based on primary/secondary and tertiary maternal education, pooled analyses revealed that adolescents of tertiary educated mothers were less active (approximately 40 min less light activity and approximately 7 min less MVPA/day), and more sedentary (approximately 46 min/day), when compared with adolescents of primary/secondary educated mothers. Although 7 min more MVPA per day by children of the primary educated mothers does not appear behaviourally significant, when considering the relatively low time spent in MVPA in this sample, the difference is quite large (approximately 25% of their total MVPA average). The lack of significance in PA and/or sedentary time between children of college educated mothers and the other groups (ie, high school and university) is somewhat to be expected. There was likely little discriminatory difference in education between these neighbouring categories, as a mother could be placed into a higher category based on a short attendance at higher education. For example, a mother who completed a few weeks on a vocational course and then withdrew would still be placed in the ‘college’ category.

Results from the present work support a small number of studies from developed countries that have used objective measurements of PA and total time sedentary to explore the association with SEP. The Gateshead Millennium Study in England found an inverse association between SEP and children's PA^22^ and The Health Survey for England (HSE) showed that children grouped into the high SEP category spent approximately 17 more minutes sedentary per day.^15^ Likewise, a previous analysis^23^ from EYHS found a positive association between children's sedentary time and SEP in Portuguese and Estonian samples, but not in the Danish and Norwegian. The results from this study contrast evidence based on self-reported PA and/or sedentary behaviours. Previous data suggest that children of low SEP report greater engagement in sedentary pursuits such as TV watching and other screen-based activities.^5^ ^10^ ^15–18^ This supports the notion that self-reporting tools and objective measurement of overall PA are not measuring the same construct. In fact within the same sample there appears to be a different association between SEP and self-reporting sedentary behaviours and objectively measured sedentary time. The aforementioned paper from the HSE^15^ revealed that low SEP was associated with high television watching, but lower total accelerometer assessed sedentary time. Likewise, although the association with total PA and SEP is inconsistent,^17^ ^19^ ^22^ studies have shown that children of higher SEP participate in more self-reported organised sports.^18^ ^33^ Whereas, two separate studies of Brazilian adolescents indicated that lower SEP youth were more likely to self-report greater PA levels and, in particular, active transport.^34^ ^35^ These studies suggest that how children from different SEPs accrue sedentary time and PA may be different.

Maternal education was chosen as the SEP indicator in the present pooled analysis because it was the most common indicator across the studies. The wider adoption of maternal education as an indicator of SEP by contributing studies reflects the fact that it is relatively easy to measure and garners a high response rate. Although composite measures of SEP often show stronger associations with health outcomes and behaviours,^36^ conclusions based on univariate indicators, such as maternal education, may offer clearer direction for policymakers. However, although maternal education is a strong determinant of parental employment and income^37^ the meaning of maternal education levels likely varies between countries, and potentially populations within a country, where education can be either compulsory or a choice. Furthermore, mothers play different cultural and societal roles depending on the country/population, while in some cultures paternal education may be more important.

The data in ICAD comes from middle-high income/developed countries with generally high national levels of education. Of the 10 studies included, 8 were from developed countries and were ranked in the top 50% for tertiary education, with two (Brazil and Portugal) in the bottom 50%.^38^ Association between PA and SEP may be dependent on the level of development of the country.^3^ For example, in developing countries, an active lifestyle may be a necessity for those in lower SEPs, whereas in developed countries, technological advances have resulted in an erosion of lifestyle-embedded PA across all social strata, and a healthy ‘active’ lifestyle may require more deliberate effort. However, evidence from developing countries in rapid transition appear to support the findings from this study. For example, in rural South Africa, adolescents whose mothers had a secondary school education or higher, reported significantly more self-reported sedentary time and significantly less walking for transport than children whose mothers had no education.^39^ In the older group, however, higher maternal education was associated with nearly 3 h more school and club MVPA per week.^39^ Thus, future research needs to explore, across countries of varying development, the association between SEP and specific behaviours, such as active commuting, technology-driven sedentary pursuits, PE participation, sports participation, etc. Further effort is also needed to determine the impact of these behaviours on overall objectively measured PA and time spent sedentary, and how they relate to health outcomes to inform intervention and policy changes.

### Strengths and limitations

Strengths include the large sample size and the meta-analysis of 10 individual studies, providing more robust estimates of observed associations. Physical activity and time spent sedentary were objectively measured, reducing possible misclassification, and raw data files were cleaned, processed and analysed in a standardised manner.^24^ Limitations include the lack of data from low income/developing countries, the cross-sectional nature of the data and lack of consideration of important potential mediators (eg, time spent in after-school sports clubs, birth weight and birth order).^22^ Our intensity threshold for MVPA (3000 CPM) was higher compared with some previous studies in youths.^40^ However, when reanalysing our data using a lower threshold, the observations were materially unchanged (data not shown). Of the 10 studies included in the analyses, only one study (Project TAAG) relied on adolescent, rather than parent-reported maternal education. It is likely that adolescent-reported maternal education is less accurate than parental reported; however, validity studies have shown fair agreement between the two.^41^ Some previous studies examining the association between PA/sedentary behaviour and maternal education have included a lower educational category (eg, primary school only, or 1–2 years of high school). Owing to original maternal educational coding used, this was not possible in the pooled analyses. This reduces the ability to directly compare our results with some previous studies. Last, maternal education was the sole SEP indicator considered, and it is likely that other measures of SEP have a different association with youth PA and sedentary behaviour.

## Conclusion

Greater maternal education appears to be associated with lower objectively assessed PA and increased time spent sedentary in adolescents from developed countries. From a public health perspective, these results are a potential good news story as they highlight that children from low SEP may not be disadvantaged in terms of overall daily PA. Future work pooling standardised accelerometer data across countries needs to prioritise the inclusion of lower income/developing countries to fully understand the association between maternal education and PA/time spent sedentary.

**What is already known on this subject**

- Socioeconomic differences in physical activity and time spent sedentary may be a mechanism linking socioeconomic position and ill health. Studies that have investigated the relationship between socioeconomic position and physical activity and sedentary behaviour have reported inconsistent findings.

**What this study adds**

- In a pooled analysis of 12 770 youths across 10 studies from Europe, Australia, Brazil and the USA, those children whose mothers reported a higher level of education (proxy for socioeconomic position (SEP)) had lower levels of objectively measured physical activity and greater time spent sedentary. This finding suggests that children from low SEP may not be disadvantaged in terms of overall daily physical activity. Future research needs to explore, across countries of varying development, the association between SEP and specific behaviours, such as active commuting, technology-driven sedentary pursuits and sports participation, and the impact these have on overall objectively measured physical activity and time spent sedentary.

## Supplementary Material

Web supplement

Web figures

## References

[1] Borrell LN, Diez Roux AV, Rose K, et al Neighbourhood characteristics and mortality in the Atherosclerosis Risk in Communities Study. Int J Epidemiol 2004;33:398–407. 10.1093/ije/dyh063 15082648

[2] Pollitt RA, Kaufman JS, Rose KM, et al Early-life and adult socioeconomic status and inflammatory risk markers in adulthood. Eur J Epidemiol 2007;22:55–66. 10.1007/s10654-006-9082-1 17225957

[3] World Health Organization. Global status report on noncommunicable diseases. Geneva: World Health Organization, 2011.

[4] Hosseinpoor AR, Bergen N, Kunst A, et al Socioeconomic inequalities in risk factors for non communicable diseases in low-income and middle-income countries: results from the World Health Survey. BMC Public Health 2012;12:912 10.1186/1471-2458-12-912 23102008 PMC3507902

[5] Lioret S, Maire B, Volatier JL, et al Child overweight in France and its relationship with physical activity, sedentary behaviour and socioeconomic status. Eur J Clin Nutr 2007;61:509–16. 16988644 10.1038/sj.ejcn.1602538

[6] Janssen I, Boyce WF, Simpson K, et al Influence of individual- and area-level measures of socioeconomic status on obesity, unhealthy eating, and physical inactivity in Canadian adolescents. Am J Clin Nutr 2006;83:139–45. 16400062 10.1093/ajcn/83.1.139

[7] Kristensen PL, Wedderkopp N, Moller NC, et al Tracking and prevalence of cardiovascular disease risk factors across socio-economic classes: a longitudinal substudy of the European youth heart study. BMC Public Health 2006;6:20 10.1186/1471-2458-6-20 16441892 PMC1403767

[8] Huurre T, Aro H, Rahkonen O Well-being and health behaviour by parental socioeconomic status: a follow-up study of adolescents aged 16 until age 32 years. Soc Psychiatry Psychiatr Epidemiol 2003;38:249–55. 10.1007/s00127-003-0630-7 12719840

[9] Lawlor DA, Harro M, Wedderkopp N, et al Association of socioeconomic position with insulin resistance among children from Denmark, Estonia, and Portugal: cross-sectional study. BMJ 2005;331:183 10.1136/bmj.331.7510.183 16037446 PMC1179757

[10] Brodersen NH, Steptoe A, Boniface DR, et al Trends in physical activity and sedentary behaviour in adolescence: ethnic and socioeconomic differences. Br J Sports Med 2007;41:140–4. 10.1136/bjsm.2006.031138 17178773 PMC2465219

[11] Stalsberg R, Pedersen AV Effects of socioeconomic status on the physical activity in adolescents: a systematic review of the evidence. Scand J Med Sci Sports 2010;20:368–83. 10.1111/j.1600-0838.2009.01047.x 20136763

[12] Sallis JF, Prochaska JJ, Taylor WC A review of correlates of physical activity of children and adolescents. Med Sci Sports Exerc 2000;32:963–75. 10.1097/00005768-200005000-00014 10795788

[13] Van Der Horst K, Paw MJ, Twisk JW, et al A brief review on correlates of physical activity and sedentariness in youth. Med Sci Sports Exerc 2007;39:1241–50. 10.1249/mss.0b013e318059bf35 17762356

[14] Shephard RJ Limits to the measurement of habitual physical activity by questionnaires. Br J Sports Med 2003;37:197–206. 10.1136/bjsm.37.3.197 12782543 PMC1724653

[15] Coombs N, Shelton N, Rowlands A, et al Children's and adolescents’ sedentary behaviour in relation to socioeconomic position. J Epidemiol Community Health 2013;67:868–74. 10.1136/jech-2013-202609 23851152 PMC3835391

[16] Hoyos Cillero I, Jago R Sociodemographic and home environment predictors of screen viewing among Spanish school children. J Public Health (Oxf) 2011;33:392–402. 10.1093/pubmed/fdq087 21047871 PMC3307230

[17] Drenowatz C, Eisenmann JC, Pfeiffer KA, et al Influence of socio-economic status on habitual physical activity and sedentary behavior in 8- to 11-year-old children. BMC Public Health 2010;10:214 10.1186/1471-2458-10-214 20423487 PMC2873582

[18] Fairclough SJ, Boddy LM, Hackett AF, et al Associations between children's socioeconomic status, weight status, and sex, with screen-based sedentary behaviours and sport participation. Int J Pediatr Obes 2009;4:299–305. 10.3109/17477160902811215 19922045

[19] McMinn AM, Griffin SJ, Jones AP, et al Family and home influences on children's after-school and weekend physical activity. Eur J Public Health 2013;23:805–10. 10.1093/eurpub/cks160 23172732 PMC3784797

[20] Kelly LA, Reilly JJ, Fisher A, et al Effect of socioeconomic status on objectively measured physical activity. Arch Dis Child 2006;91:35–8. 10.1136/adc.2005.080275 16239246 PMC2083107

[21] Ball K, Cleland VJ, Timperio AF, et al Socioeconomic position and children's physical activity and sedentary behaviors: longitudinal findings from the CLAN study. J Phys Act Health 2009;6:289–98. 19564656 10.1123/jpah.6.3.289

[22] Pearce MS, Basterfield L, Mann KD, et al Early predictors of objectively measured physical activity and sedentary behaviour in 8–10-year-old children: the Gateshead Millennium Study. PLoS ONE 2012;7:e37975 10.1371/journal.pone.0037975 22745660 PMC3380043

[23] van Sluijs EM, Page A, Ommundsen Y, et al Behavioural and social correlates of sedentary time in young people. Br J Sports Med 2010;44:747–55. 10.1136/bjsm.2008.049783 18812418

[24] Sherar LB, Griew P, Esliger DW, et al International children's accelerometry database (ICAD): design and methods. BMC Public Health 2011;11:485 10.1186/1471-2458-11-485 21693008 PMC3146860

[25] Troiano RP, Berrigan D, Dodd KW, et al Physical activity in the United States measured by accelerometer. Med Sci Sports Exerc 2008;40:181–8. 10.1249/mss.0b013e31815a51b3 18091006

[26] Ekelund U, Fau-Luan J, Luan J, et al Moderate to vigorous physical activity and sedentary time and cardiometabolic risk factors in children and adolescents. JAMA 2012;307:704–12. (1538–3598 (Electronic); 0098–7484 (Linking)) 10.1001/jama.2012.156 22337681 PMC3793121

[27] Toftager M, Kristensen PL, Oliver M, et al Accelerometer data reduction in adolescents: effects on sample retention and bias. Int J Behav Nutr Phys Act 2013;10:140 10.1186/1479-5868-10-140 24359480 PMC3880051

[28] Treuth MS, Schmitz K, Catellier DJ, et al Defining accelerometer thresholds for activity intensities in adolescent girls. Med Sci Sports Exerc 2004;36: 1259–66. 10.1249/01.MSS.0000113666.98463.B0 15235335 PMC2423321

[29] Mattocks C, Leary S, Ness A, et al Calibration of an accelerometer during free-living activities in children. Int J Pediatr Obes 2007;2:218–26. 10.1080/17477160701408809 17852552

[30] Cole TJ, Bellizzi MC, Flegal KM, et al Establishing a standard definition for child overweight and obesity worldwide: international survey. BMJ 2000;320: 1240–3. 10.1136/bmj.320.7244.1240 10797032 PMC27365

[31] Higgins JP, Thompson SG Quantifying heterogeneity in a meta-analysis. Stat Med 2002;21:1539–58. 10.1002/sim.1186 12111919

[32] Higgins JP, Thompson SG, Deeks JJ, et al Measuring inconsistency in meta-analyses. BMJ 2003;327:557–60. 10.1136/bmj.327.7414.557 12958120 PMC192859

[33] Wichstrom L, von Soest T, Kvalem IL Predictors of growth and decline in leisure time physical activity from adolescence to adulthood. Health Psychol 2013;32:775–84. 10.1037/a0029465 22924445

[34] Alves CF, Silva R de C, Assis AM, et al Factors associated with physical inactivity in adolescents aged 10–14 years, enrolled in the public school network of the city of Salvador, Brazil. Rev Bras Epidemiol 2012;15:858–70. 10.1590/S1415-790X2012000400016 23515780

[35] Hallal PC, Wells JC, Reichert FF, et al Early determinants of physical activity in adolescence: prospective birth cohort study. BMJ 2006;332:1002–7. 10.1136/bmj.38776.434560.7C 16601016 PMC1450043

[36] Oakes M Measuring socioeconomic status. http://www.esourceresearch.org/Portals/0/Uploads/Documents/Public/Oakes_FullChapter.pdf (accessed 18 Jul 2013).

[37] Davey Smith G, Hart C, Hole D, et al Education and occupational social class: which is the more important indicator of mortality risk? J Epidemiol Community Health 1998;52:153–60. 10.1136/jech.52.3.153 9616419 PMC1756692

[38] OECD. http://www.oecd.org/edu/eag2012 (accessed 22 Oct 2012).

[39] Micklesfield LK, Pedro TM, Kahn K, et al Physical activity and sedentary behavior among adolescents in rural South Africa: levels, patterns and correlates. BMC Public Health 2014;14:40 10.1186/1471-2458-14-40 24433276 PMC3897951

[40] Andersen LB, Harro M, Sardinha LB, et al Physical activity and clustered cardiovascular risk in children: a cross-sectional study (The European Youth Heart Study). Lancet 2006;368:299–304. 10.1016/S0140-6736(06)69075-2 16860699

[41] Lien N, Friestad C, Klepp KI Adolescents’ proxy reports of parents’ socioeconomic status: how valid are they? J Epidemiol Community Health 2001;55:731–7. 10.1136/jech.55.10.731 11553657 PMC1731778

