# Supplementary material for: Association between maternal education and objectively measured physical activity and sedentary time in adolescents
Source: J Epidemiol Community Health. 2016 Jan 22;70(6):541–8. doi: 10.1136/jech-2015-205763 (PMC4893139; doi:10.1136/jech-2015-205763)
Supplement: Web supplement [file jech-2015-205763-s1.pdf]

**Table S1: Raw education categories underlying re-coded maternal education data**

| Study | Raw maternal education categories | Re-coded categories |
|---|---|---|
| Denmark EYHS | • Primary<br>• Secondary<br>• EFG basic<br>• EFG 2 | Up to high school |
| | • Apprentice<br>• Short further education | College / vocational |
| | • Medium or long-term further education | University level |
| Estonia EYHS | • < 8 years (basic education)<br>• 2- 11 years (secondary education) | Up to high school |
| | • 11-13 years (specialised secondary) | College / vocational |
| | • Higher not graduated<br>• Higher education | University level |
| Switzerland KISS | • Mandatory schooling only<br>• Apprenticeship/informal education of under 2 years | Up to high school |
| | • Formal apprenticeship of 2-4 years | College / vocational |
| | • University or equivalent | University level |
| ALSPAC | • No qualifications<br>• O-levels, CSEs or GCSEs<br>• A-levels<br>• Qualifications in shorthand, typing or other skills | Up to high school |
| | • Apprenticeship<br>• State enrolled nurse or state registered nurse<br>• City & Guilds intermediate or final technical qualification | College / vocational |
| | • Teaching qualification<br>• University degree | University level |
| PEACH | • GCSE<br>• A-level | Up to high school |
| | • First degree<br>• Higher degree | University level |
| CLAN and HEAPS | • Never attended school<br>• Primary school<br>• Some high school<br>• Completed high school | Up to high school |
| | • Technical or trade school certificate/ apprenticeship | College / vocational |
| | • University or tertiary qualification | University level |
| Project TAAG | • Did not finish high school<br>• Finished high school | Up to high school |
| | • Vocational training<br>• Some college | College / vocational |
| | • Graduated college or university<br>• Professional training beyond 4 years | University level |
| Madeira EYHS | • No school<br>• 1st grade through to 10th grade | Up to high school |
| | • 11 or 12th grade | College / vocational |
| Pelotas 1993 Birth Cohort | • No school<br>• 1st grade through to 10th grade | Up to high school |
| | • 11 or 12th grade | College / vocational |

**SUPPLEMENTARY INFORMATION**

**Descriptions of the ALSPAC study**

## Avon Longitudinal Study of Parents and Children

ALSPAC recruited pregnant women resident in Avon, UK with expected dates of delivery 1st April 1991 to 31st December 1992. In approximately 1998, the initial sample was expanded by recruiting eligible cases who did not join the study originally. Further details are published previously [1]. The total sample size for analyses using any data collected after the age of seven is from 15,247 pregnancies, resulting in 14,701 children alive at 1 year of age. Samples for the studies reported here were obtained from 1,567 for participants who had attended before 1100h. All the data are available through a fully searchable data dictionary at http://www.bris.ac.uk/alspac/researchers/data-access/data-dictionary. Ethical approval for the study was obtained from the ALSPAC Ethics and Law Committee and the Local Research Ethics Committees. Plasma cortisol was measured by RIA (MP Biomedicals, Cambridge, UK).

1. Boyd A, Golding J, Macleod J, Lawlor DA, Fraser A et al. (2013) Cohort Profile: The 'Children of the 90s'--the index offspring of the Avon Longitudinal Study of Parents and Children. Int J Epidemiol 42: 111-127.
