# Supplementary material for: Association between maternal education and objectively measured physical activity and sedentary time in adolescents
Source: J Epidemiol Community Health. 2016 Jan 22;70(6):541–8. doi: 10.1136/jech-2015-205763 (PMC4893139; doi:10.1136/jech-2015-205763)
Supplement: Web figures [file jech-2015-205763-s2.pdf]

# Supplementary Figures

Supplementary Figure S1: Forest plot showing mean differences, before adjustment for BMI status, in physical activity variables between studies where maternal education defined as: high school (reference group), college and university.

Supplementary Figure S2: Forest plot showing mean differences, before adjustment for BMI status, in physical activity variables between studies where maternal education defined as: primary (reference group), secondary and tertiary.

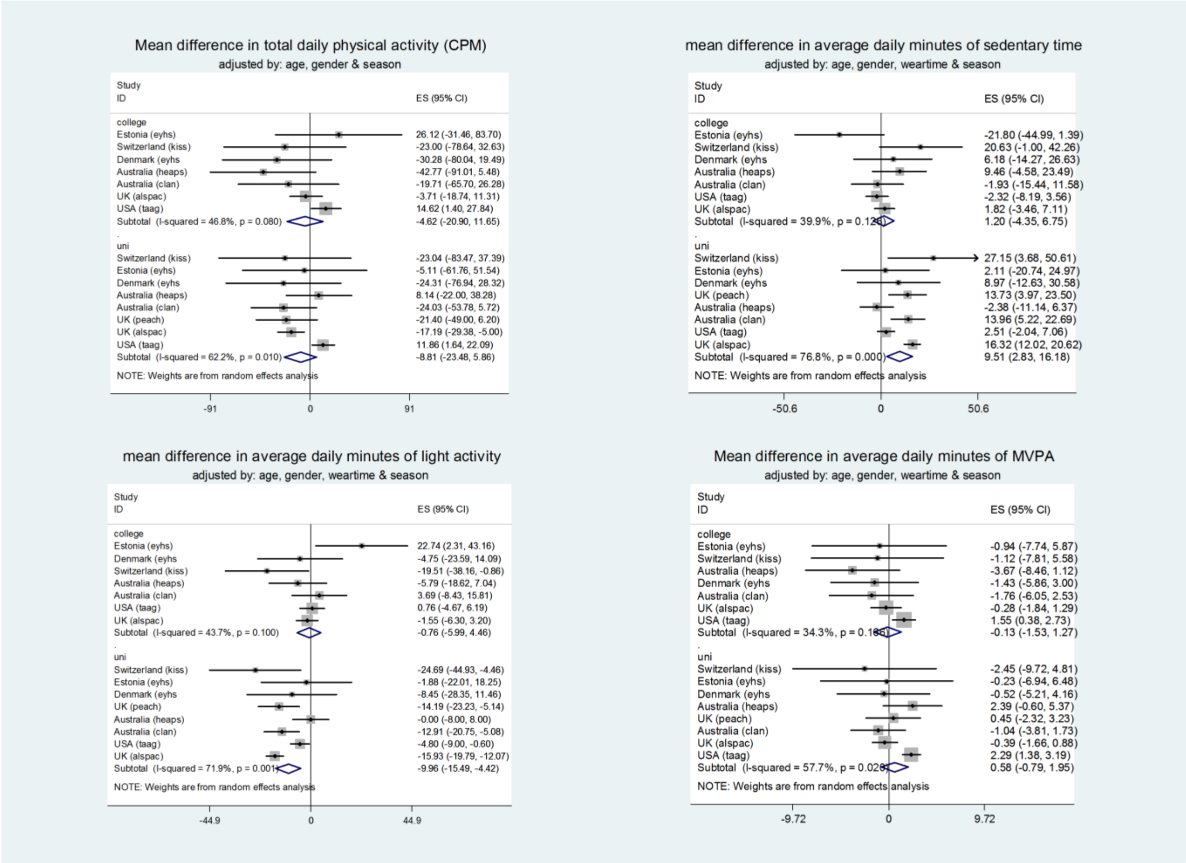
Mean difference in total daily physical activity (CPM)
adjusted by: age, gender & season
Study ID
ES (95% CI)
college
Estonia (eyhs) 26.12 (-31.46, 83.70)
Switzerland (kiss) -23.00 (-78.64, 32.63)
Denmark (eyhs -30.28 (-80.04, 19.49)
Australia (heaps) -42.77 (-91.01, 5.48)
Australia (clan) -19.71 (-65.70, 26.28)
UK (alspac) -3.71 (-18.74, 11.31)
USA (taag) 14.62 (1.40, 27.84)
Subtotal (I-squared = 46.8%, p = 0.080) -4.62 (-20.90, 11.65)
uni
Switzerland (kiss) -23.04 (-83.47, 37.39)
Estonia (eyhs) -5.11 (-61.76, 51.54)
Denmark (eyhs -24.31 (-76.94, 28.32)
Australia (heaps) 8.14 (-22.00, 38.28)
Australia (clan) -24.03 (-53.78, 5.72)
UK (peach) -21.40 (-49.00, 6.20)
UK (alspac) -17.19 (-29.38, -5.00)
USA (taag) 11.86 (1.64, 22.09)
Subtotal (I-squared = 62.2%, p = 0.010) -8.81 (-23.48, 5.86)
NOTE: Weights are from random effects analysis
-91
0
91
mean difference in average daily minutes of sedentary time
adjusted by: age, gender, weartime & season
Study ID
ES (95% CI)
college
Estonia (eyhs) -21.80 (-44.99, 1.39)
Switzerland (kiss) 20.63 (-1.00, 42.26)
Denmark (eyhs 6.18 (-14.27, 26.63)
Australia (heaps) 9.46 (-4.58, 23.49)
Australia (clan) -1.93 (-15.44, 11.58)
USA (taag) -2.32 (-8.19, 3.56)
UK (alspac) 1.82 (-3.46, 7.11)
Subtotal (I-squared = 39.9%, p = 0.126) 1.20 (-4.35, 6.75)
uni
Switzerland (kiss) 27.15 (3.68, 50.61)
Estonia (eyhs) 2.11 (-20.74, 24.97)
Denmark (eyhs 8.97 (-12.63, 30.58)
UK (peach) 13.73 (3.97, 23.50)
Australia (heaps) -2.38 (-11.14, 6.37)
Australia (clan) 13.96 (5.22, 22.69)
USA (taag) 2.51 (-2.04, 7.06)
UK (alspac) 16.32 (12.02, 20.62)
Subtotal (I-squared = 76.8%, p = 0.000) 9.51 (2.83, 16.18)
NOTE: Weights are from random effects analysis
-50.6
0
50.6
mean difference in average daily minutes of light activity
adjusted by: age, gender, weartime & season
Study ID
ES (95% CI)
college
Estonia (eyhs) 22.74 (2.31, 43.16)
Denmark (eyhs -4.75 (-23.59, 14.09)
Switzerland (kiss) -19.51 (-38.16, -0.86)
Australia (heaps) -5.79 (-18.62, 7.04)
Australia (clan) 3.69 (-8.43, 15.81)
USA (taag) 0.76 (-4.67, 6.19)
UK (alspac) -1.55 (-6.30, 3.20)
Subtotal (I-squared = 43.7%, p = 0.100) -0.76 (-5.99, 4.46)
uni
Switzerland (kiss) -24.69 (-44.93, -4.46)
Estonia (eyhs) -1.88 (-22.01, 18.25)
Denmark (eyhs -8.45 (-28.35, 11.46)
UK (peach) -14.19 (-23.23, -5.14)
Australia (heaps) -0.00 (-8.00, 8.00)
Australia (clan) -12.91 (-20.75, -5.08)
USA (taag) -4.80 (-9.00, -0.60)
UK (alspac) -15.93 (-19.79, -12.07)
Subtotal (I-squared = 71.9%, p = 0.001) -9.96 (-15.49, -4.42)
NOTE: Weights are from random effects analysis
-44.9
0
44.9
Mean difference in average daily minutes of MVPA
adjusted by: age, gender, weartime & season
Study ID
ES (95% CI)
college
Estonia (eyhs) -0.94 (-7.74, 5.87)
Switzerland (kiss) -1.12 (-7.81, 5.58)
Australia (heaps) -3.67 (-8.46, 1.12)
Denmark (eyhs -1.43 (-5.86, 3.00)
Australia (clan) -1.76 (-6.05, 2.53)
UK (alspac) -0.28 (-1.84, 1.29)
USA (taag) 1.55 (0.38, 2.73)
Subtotal (I-squared = 34.3%, p = 0.165) -0.13 (-1.53, 1.27)
uni
Switzerland (kiss) -2.45 (-9.72, 4.81)
Estonia (eyhs) -0.23 (-6.94, 6.48)
Denmark (eyhs -0.52 (-5.21, 4.16)
Australia (heaps) 2.39 (-0.60, 5.37)
UK (peach) 0.45 (-2.32, 3.23)
Australia (clan) -1.04 (-3.81, 1.73)
UK (alspac) -0.39 (-1.66, 0.88)
USA (taag) 2.29 (1.38, 3.19)
Subtotal (I-squared = 57.7%, p = 0.028) 0.58 (-0.79, 1.95)
NOTE: Weights are from random effects analysis
-9.72
0
9.72

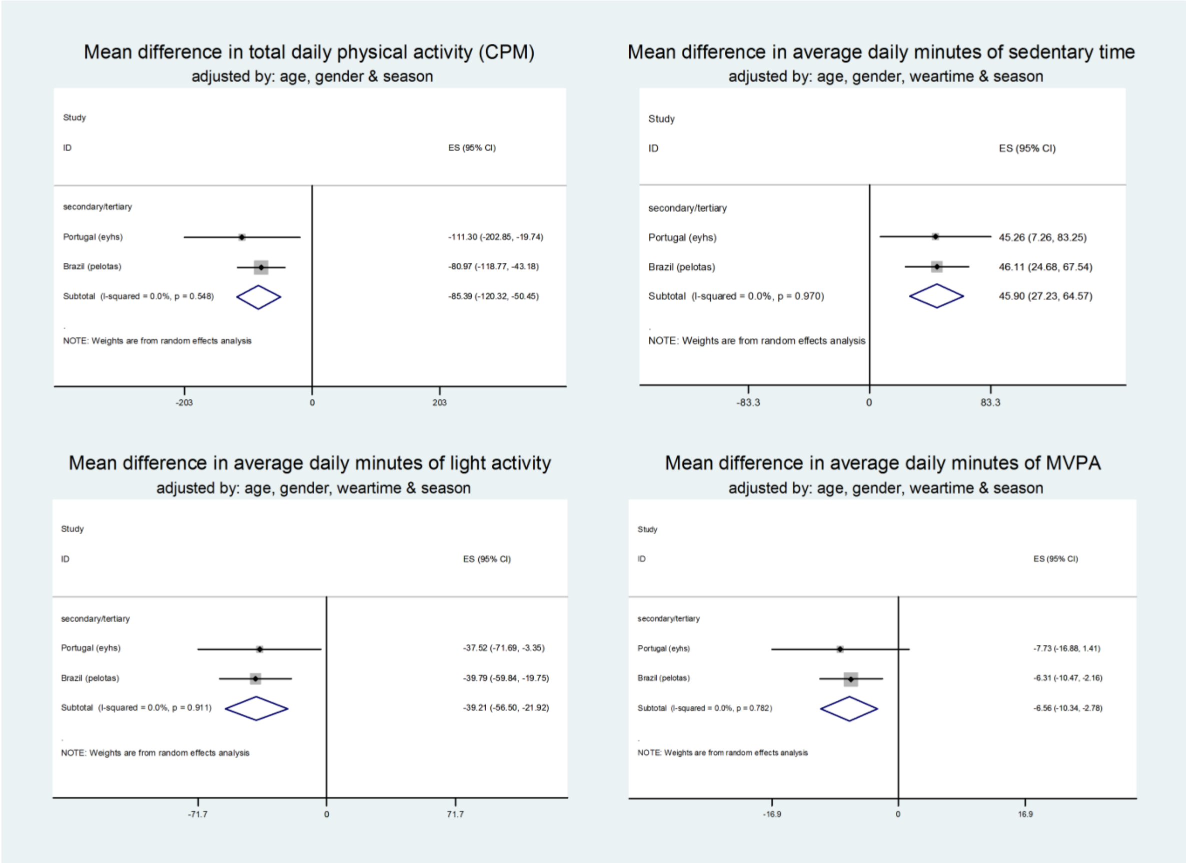
Mean difference in total daily physical activity (CPM)
adjusted by: age, gender & season
Study ID
ES (95% CI)
secondary/tertiary
Portugal (eyhs) -111.30 (-202.85, -19.74)
Brazil (pelotas) -80.97 (-118.77, -43.18)
Subtotal (I-squared = 0.0%, p = 0.548) -85.39 (-120.32, -50.45)
NOTE: Weights are from random effects analysis
-203
0
203
Mean difference in average daily minutes of sedentary time
adjusted by: age, gender, weartime & season
Study ID
ES (95% CI)
secondary/tertiary
Portugal (eyhs) 45.26 (7.26, 83.25)
Brazil (pelotas) 46.11 (24.68, 67.54)
Subtotal (I-squared = 0.0%, p = 0.970) 45.90 (27.23, 64.57)
NOTE: Weights are from random effects analysis
-83.3
0
83.3
Mean difference in average daily minutes of light activity
adjusted by: age, gender, weartime & season
Study ID
ES (95% CI)
secondary/tertiary
Portugal (eyhs) -37.52 (-71.69, -3.35)
Brazil (pelotas) -39.79 (-59.84, -19.75)
Subtotal (I-squared = 0.0%, p = 0.911) -39.21 (-56.50, -21.92)
NOTE: Weights are from random effects analysis
-71.7
0
71.7
Mean difference in average daily minutes of MVPA
adjusted by: age, gender, weartime & season
Study ID
ES (95% CI)
secondary/tertiary
Portugal (eyhs) -7.73 (-16.88, 1.41)
Brazil (pelotas) -6.31 (-10.47, -2.16)
Subtotal (I-squared = 0.0%, p = 0.782) -6.56 (-10.34, -2.78)
NOTE: Weights are from random effects analysis
-16.9
0
16.9
